# Long-term mortality among patients with community acquired severe sepsis and septic shock

**DOI:** 10.1186/1757-7241-20-S2-P28

**Published:** 2012-04-16

**Authors:** Merete Storgaard, Jesper Hallas, Bente Gahrn-Hansen, Svend Stenvang Pedersen, Court K Pedersen, Annmarie Lassen

**Affiliations:** 1 Department of Infectious Diseases, University Hospital Aarhus, Skejby, Denmark; 2 Department of Clinical Pharmacology, University of South Denmark, Denmark; 3 Department of Clinical Microbiology, Odense University Hospital, Denmark; 4 Department of Infectious Diseases, Odense University Hospital, Denmark; 5 Department of Infectious Diseases, Odense University Hospital, Denmark; 6 Department of Emergency Medicine, Odense University Hospital, Denmark

## Background

Severe sepsis and septic shock has a high one month mortality (10%-50%) but long term mortality is scarcely described.

## Aim

To describe long term mortality among patients with community acquired severe sepsis or septic shock compared to a population based reference cohort.

## Methods

Patients with a first time discharge diagnosis of sepsis (IDC10 code A40.0 – A41.9) were identified from the Funen Patient Administrative System. Clinical details were evaluated manually and patients who within the first 24 hours after arrival to the hospital presented with infection and had failure of minimum one organ system were included.

A population based reference group on 79,857 patients were identified.

Data on co-morbidity were obtained from the National Danish Patient Registry.

## Results

335 patients were identified by discharge diagnosis. 212 had infection at arrival to the hospital and failure of minimum one organ system – 50% males, mean age 70.6 years (SD 14.7 range 24.0-96.6 years), 103 (49%) had septic shock.

Follow up was possible for 211. Median follow up time was 1.9 years (range 0.0 – 5.0 years). Mortality within the first month was 69/211 (33%, 95% CI 25-41 %), with a cumulative mortality of 121/211 (57%, 95% CI 48-69%) for the complete follow up.

Given the patient survived the first month, mortality until day 365 was 158/1000 person years (95% CI 120-248). Given the patient survived the first year mortality from year 1 to year 4 was 143/1000 person years (95% CI 98-200).

Among septic patients who survived the first month mortality hazard ratio was 2.7 (95% CI 1.7-4.3) until day 365, and among septic patients who survived the first year, the one year to four years mortality hazard ratio was 2.3 (95% CI 1.7-3.3) compared to community based reference persons (multivariate cox regression controlling for age, sex and co-morbidity).

## Conclusion

Patients with severe sepsis and septic shock who survived the first month have a 2.7 times higher mortality rate the first year and a 2.3 times higher mortality rate the next three years compared to persons with similar age, sex and co-morbidity.

